# Remote cognitive rehabilitation of attention: a case series pilot study with post-stroke patients

**DOI:** 10.1590/1980-5764-DN-2023-0045

**Published:** 2023-12-15

**Authors:** Letícia Silva Dutra, Larissa Scoralich, Nadia Shigaeff

**Affiliations:** 1 Universidade Federal de Juiz de Fora, Grupo Interdisciplinar de Pesquisa em Neuropsicologia e Gerontologia, Juiz de Fora MG, Brazil.

**Keywords:** Stroke, Cognitive Training, Attention, Acidente Vascular Cerebral, Treino Cognitivo, Atenção

## Abstract

Cognitive deficits are highly prevalent following a stroke, with memory, attention, and psychomotor speed being among the most affected functions. Prior research indicates that cognitive rehabilitation for stroke patients yields substantial improvements in cognitive performance and concurrently exerts a positive influence on reducing depressive symptoms.

**Objective:**

The goal of this study was to provide a description and assessment of the influence of cognitive rehabilitation on the attentional performance and depressive symptoms of individuals diagnosed with stroke.

**Methods:**

Participants underwent a neuropsychological assessment both prior to and following a 15-week remote cognitive rehabilitation intervention. This intervention involved the implementation of various cognitive tasks aimed at rehabilitating attentional skills.

**Results:**

The outcomes of the individualized descriptive assessment revealed a no table inclination towards enhanced attentional performance. The comparative results indicated that the cognitive rehabilitation intervention for stroke patients proved effective in facilitating a substantial reduction in depressive symptoms and enhancing participants’ alternating attention. While it is acknowledged that certain individuals may still exhibit deficiencies in various facets of attentional performance, cognitive rehabilitation contributed to the clinical amelioration of these individuals.

**Conclusion:**

Clinical improvement holds profound significance in the day-to-day existence of these individuals, bolstering their autonomy and fortifying their sense of self-efficacy, as attested by their personal perceptions and self-reports.

## RESUMO

Os déficits cognitivos são altamente prevalentes após um acidente vascular cerebral (AVC), sendo a memória, a atenção e a velocidade psicomotora algumas das funções mais afetadas. Pesquisas anteriores demonstram que a reabilitação cognitiva em pacientes com AVC resulta em melhorias substanciais no desempenho cognitivo e exerce simultaneamente uma influência positiva na redução dos sintomas depressivos.

**Objetivo:**

O objetivo deste estudo foi fornecer uma descrição e avaliação da influência da reabilitação cognitiva no desempenho da atenção e de sintomas depressivos de indivíduos diagnosticados com AVC.

**Métodos:**

Os participantes foram submetidos a uma avaliação neuropsicológica antes e depois de uma intervenção de reabilitação cognitiva remota de 15 semanas. Essa intervenção envolveu a implementação de várias tarefas cognitivas destinadas à reabilitação das habilidades de atenção.

**Resultados:**

Os resultados da avaliação descritiva individualizada revelaram notável tendência para o aprimoramento do desempenho da atenção. Os resultados comparativos revelaram que a intervenção de reabilitação cognitiva para pacientes com AVC se mostrou eficaz na facilitação de uma redução substancial nos sintomas depressivos e no aprimoramento da atenção alternada dos participantes. Embora seja reconhecido que certos indivíduos ainda podem apresentar prejuízo no desempenho atencional, a reabilitação cognitiva contribuiu para a melhora clínica desses indivíduos.

**Conclusão:**

A melhora clínica possui profunda significância na existência cotidiana desses indivíduos, fortalecendo sua autonomia e reforçando sua sensação de autoeficácia, como atestado por suas percepções pessoais e relatos próprios.

## INTRODUCTION

A cerebral vascular accident or stroke is associated with a high degree of morbidity and mortality, and, if it does not result in death, it can lead to significant functional disability in those who suffer it^1^. The most common impairments are observed in motor, cognitive, emotional and behavioral performance, depending on the affected area, and can be temporary or permanent.

There are few publications on remote neuropsychological rehabilitation, especially in patients with cerebrovascular injuries. The majority of research endeavors pertain to language rehabilitation, and the utilization of virtual environments is a prevalent practice^2^. Additionally, the limited number of studies focusing on attentional performance within the population afflicted by brain injuries may be attributed to challenges associated with delineating the concept of attention, coupled with the absence of a consensus on standardized measurement methodologies^3^.

The most recent theoretical models in neuropsychology characterize attention based on how it is operationalized: selective, sustained, alternating and divided. Selective attention is the individual’s ability to privilege certain stimuli to the detriment of others. Sustained attention describes the individual’s ability to maintain attentional focus on a given stimulus or sequence of stimuli during a period of time for the performance of a task. Alternate attention is the individual’s ability to alternate attentional focus, alternately deconcentrating from one focus and concentrating on another. And, finally, divided attention is characterized by the performance of two tasks simultaneously^4,5^.

Neuropsychiatric complications frequently manifest following a stroke^6,7^, with the most prevalent among them being post-stroke depression (PSD), impacting a range of 5 to 75% of patients^8^. Furthermore, PSD has been found to be linked with cognitive impairments in areas encompassing memory and attention, as well as reduced psychomotor speed^9–11^. Additionally, these complications can be associated with observable constraints in daily activities^6,7^ and hindered functional recovery^12^.

To restore impaired cognitive functions, it is imperative to formulate tailored therapeutic strategies targeting the particular deficits of individual patients, with the goal of rejuvenating or stimulating their functional and cognitive abilities^13^. Prior research underscores the substantial efficacy of cognitive rehabilitation in stroke patients, yielding noteworthy enhancements in cognitive performance. Furthermore, rehabilitation interventions have been observed to mitigate depressive symptoms and enhance the autonomy of elderly individuals^14–16^.

A study pertaining to Alzheimer’s disease and vascular dementia conducted by Clare^17^ posited that cognitive stimulation, cognitive training, and cognitive rehabilitation are non-pharmacological interventions designed to optimize cognitive capacity and functional abilities in individuals with early-stage dementia. Nonetheless, the author contends that distinctions can be elucidated among these three terms. The concept of cognitive training encompasses the supervised execution of a predetermined set of standardized exercises intended to target particular cognitive functions, such as memory, attention, or problem-solving. It can be administered to a wide range of individuals, including those in good health, with the goal of preserving or enhancing cognitive performance. In contrast, cognitive stimulation endeavors to provide a broader and more generalized enhancement of cognitive performance, encompassing thinking, memory, and attention, and is typically administered within group settings. Lastly, cognitive rehabilitation pertains to the recuperation of individuals grappling with cognitive impairments stemming from illness or injury. Its objective is to facilitate the patient in attaining or preserving an “optimal minimum level of physical, psychological, and social functioning”. Typically, rehabilitation entails the implementation of personalized treatment plans with a predominant emphasis on enhancing functionality within the context of daily life, rather than solely focusing on the execution of cognitive tasks.

Recent advancements in information and communication technologies, coupled with research in the field of e-health, have brought about substantial transformations in healthcare, particularly in the aftermath of the COVID-19 pandemic and the implementation of social isolation measures. These technologies encompass various formats, such as interactive games and digital platforms, and have found utility across diverse domains, including the realm of neuropsychological rehabilitation for individuals recovering from brain injuries^18^. In spite of the increasing attention to this topic, there is a dearth of comprehensive studies focusing on stroke patients.

Consequently, the primary aim of the current investigation was to assess the impact of cognitive rehabilitation on the attentional performance and depressive symptoms of individuals diagnosed with stroke.

## METHODS

The study was structured as a pilot investigation within an open-label, single-arm clinical trial designed to assess efficacy. Consequently, this paper serves as a presentation of a case series that formed an integral component of this pilot study. The intervention was meticulously planned and executed with the objective of implementing a remote cognitive rehabilitation program aimed at enhancing the attentional performance of patients diagnosed with stroke within the past year. Pre-intervention (T1) and post-intervention (T2) neuropsychological evaluations were conducted. The neuropsychological assessments were administered in person at the Neurology Outpatient Clinic of the University Hospital of the Universidade Federal de Juiz de Fora, while the cognitive rehabilitation intervention was conducted within a virtual environment via a digital platform.

Eligible participants for this study were individuals aged 40 years or older who had received a stroke diagnosis within one year prior to the commencement of the research. Individuals who were illiterate and/or had preexisting diagnoses justifying cognitive impairment were excluded from participation in the study.

The assessment tools were administered following approval from the Research Ethics Committee for Human Research and obtaining informed, voluntary consent from all participants (Certificate of Presentation for Ethical Appreciation — CAAE: 30821220.6.0000.5147). Additionally, the study underwent evaluation, received approval, and was duly registered with the International Clinical Trials Registry Platform of the World Health Organization (ICTRP/WHO) and The Brazilian Clinical Trials Registry (Registro Brasileiro de Ensaios Clínicos — ReBEC) under the protocol RBR-10935tq4.

The instruments employed in this study comprised structured questionnaires aimed at gathering socio-demographic and clinical information, along with cognitive screening assessments using the Mini-Mental State Examination (MMSE). For the evaluation of attentional functions, the Trail Making Test (Part A and B)^19^ was administered to measure sustained and alternating attention, the Divided Attention Test (D2)^20^ was utilized to gauge divided attention, and the Stroop Color-Word Test (SCWT)^21^ was employed to appraise selective attention. Lastly, the Beck Depression Inventory-II (BDI-II)^22^ was employed to evaluate the presence of depressive symptoms.

Eligible participants were invited to undergo pre-intervention assessments encompassing socio-demographic, clinical, cognitive, and mood-related evaluations at the onset of the study. Subsequently, weekly remote sessions, each lasting 60 minutes, were conducted. During these sessions, attentional training tasks were crafted, utilizing video resources (sourced from YouTube) and exercises (such as the “7 errors task”) to address sustained attention, oral tasks (involving alphabetical and numerical ordering) for alternating attention, visual tasks (involving colors and words, inspired by the Stroop Color-Word Test) for selective attention, and exercises involving the identification of symbols to assess divided attention. The program was further tailored to accommodate specific preferences and demands of the participants. These activities were displayed on the screen for the study participants. Subsequently, individuals engaged in oral interactions and were grouped into pairs or trios. Researchers offered feedback on both successes and errors. In instances where participants made mistakes during the exercises, the researchers would reiterate the task, identify the error, and elucidate the correct method for its execution. In addition to the activities conducted during the supervised sessions, self-guided exercises were provided to the participants for ongoing training throughout the week, with progress being assessed in the subsequent session. The participants received these activities electronically and subsequently printed them out for completion using paper and pencil. At the outset of the subsequent session, attended by the participants, the researchers revisited these homework assignments and furnished feedback pertaining to both errors and accomplishments. This approach permitted participants to revisit and rectify tasks in the event of errors. Upon the completion of the fifteen-week duration of the remote cognitive rehabilitation program, cognitive and mood assessments were once again administered.

The intervention sessions were facilitated by members of the research team, with individuals being allocated to groups of two to three participants based on the extent of cognitive impairment stemming from the stroke. These groups were categorized into a mild group, characterized by relatively subtle impairments (up to 1.5 standard deviations below the population mean), and a moderate group, featuring more pronounced impairments (exceeding 1.6 standard deviations below the population mean). A parallel study employed a cognitive protocol akin to the one utilized in the current investigation^23^.

The results were presented descriptively, encompassing an analysis of the socio-demographic data and cognitive performance of each participant. Furthermore, a comparative analysis was conducted to assess changes in mood and cognitive performance before and after the intervention. All results concerning cognitive test performance were computed as z-scores or weighted, taking into account the age or education level of the participants, in accordance with the standardization criteria for each assessment tool. The z-scores for sustained, alternating, and selective attention tests were derived from the task completion time (in seconds). The weight of the divided attention test was determined by the number of correct responses provided by the participant during task execution. Despite the sample size, a comparative analysis between pre- and post-intervention performances was also carried out using the Wilcoxon Signed Ranks test. Statistically significant differences were considered to be p-value less than or equal to 0.05. All statistical analyses were performed using IBM Statistical Package for the Social Sciences (SPSS) Statistics 22.

## RESULTS

The initial sample comprised 15 participants, with a 40% dropout rate, resulting in six participants who ultimately engaged in the intervention protocol. The reasons for dropout were diverse, primarily revolving around challenges related to commuting from their residences to the hospital for test administration, limited access to the internet, mobile phones, or computers, lack of motivation, and time constraints to participate in a weekly intervention.

The ultimate sample comprised six participants, with an equal distribution of three males (50%) and three females (50%). Their ages ranged from 53 to 75 years, with a mean age of 65 years (±7.79). The mean time from diagnosis to the initial evaluation was 6.5 months (±2.58). Participants exhibited MMSE scores indicative of overall cognitive preservation, with an average score of 24 points at the study’s commencement. Importantly, this score remained consistent following the cognitive rehabilitation period, with the same mean score. These findings are summarized in Table 1.

**Table 1:** Sample demographics.

Parameters		
**Sex (n)**	Female	3
	Male	3
**Education (n)**	Elementary school graduate	1
	High school graduate	0
	University degree	1
	Postgraduate degree	0
**Age (years)**	Mean	65
	Standard deviation	(7.79)
**Marital status (n)**	Single	1
	Married	2
	Divorced	3
	Widower	0
**Diagnosis (months)**	Mean	6.5
	Standard deviation	2.58

Concerning the administration of the assessments, there were relatively few challenges encountered, and the patients demonstrated good adherence, requiring no assistance from family members. However, it is noteworthy that the D2 test, assessing divided attention, could not be administered to 50% of the participants due to visual impairments, as the test contained elements with small lettering. The cognitive performance outcomes for each participant have been delineated in Table 2.

**Table 2:** The cognitive performance results of each participant of the study.

Tests		Participant 1	Participant 2	Participant 3	Participant 4	Participant 5	Participant 6
TMT A	Pre-test	-0.94	-34.99	-12.70	-1.36	-4.06	-6.73
	Post-test	-0.68	-16.5	-7.07	0.24	-6.58	-4.63
TMT B	Pre-test	-2.17	-18.13	-9.65	-1.19	-3.45	-8.3
	Post-test	0.55	-14.13	-5.68	-0.83	-3.59	-7.24
STROOP	Pre-test	1.64	-1.92	2.28	0.17	-0.41	0.66
	Post-test	2.12	1.8	0.54	0.01	0.78	1.98
D2	Pre-test	103	58	–*	104	–*	225
	Post-test	–*	73	–*	106	–*	40

Notes:

* Some participants are unable to take the D2 test because of visual impairments

** The D2 test scores are presented in terms of the number of correct responses, while the others are expressed in z-score measurements.

Regarding the performance of individuals across each of the employed instruments, both before and after the intervention, it is noteworthy that a statistically significant improvement was observed in the context of depressive symptoms (p=0.043) and performance in alternating attention (p=0.046) (Table 3).

**Table 3:** Comparison of participants’ pre- and post-intervention cognitive performance.

Parameters	Pre-intervention Mean (SD)	Post-intervention Mean (SD)	p-value
Depressive symptomatology****	16.66 (9.45)	9.33 (7.28)	0.043*
Sustained attention**	-10.13 (12.91)	-5.87 (6.01)	0.173
Alternate attention**	-7.14 (6.35)	-5.15 (5.27)	0.046*
Divided attention***	122.5 (71.62)	73 (33)	–
Selective attention**	0.4 (1.49)	12 (0.87)	0.345

Notes:

* Statistical significance from the analysis with the Wilcoxon Test

** results presented in z-score

*** result presented in the weighted score, according to the instrument manual

**** result presented in raw score, according to the instrument manual.

## DISCUSSION

The findings of this study demonstrate the efficacy of cognitive rehabilitation intervention for stroke patients, particularly those within a one-year post-stroke timeframe. Notably, the intervention led to a significant improvement in depressive symptoms and alternate attention among the participants. This effectiveness aligns with the findings of Clare^17^, which also highlight the capacity of cognitive rehabilitation to enhance performance in neuropsychological domains, including immediate and delayed memory, working memory, attention, language, and executive function, post-intervention. Furthermore, while some individuals may still exhibit impairments in various facets of attentional functioning, it is plausible that cognitive rehabilitation has mitigated the potential for further deterioration, given the absence of worsening in attentional profiles^24^.

Except for two individuals who exhibited a decline in attentional performance in at least one domain, the majority of other participants demonstrated substantial enhancements in overall attentional performance. Remarkably, one participant exhibited such a remarkable improvement that they initially exhibited severe impairments prior to the intervention, subsequently achieving a level of performance consistent with the norms associated with cognitive functioning.

Regarding depressive symptoms, as assessed by the BDI, all participants in the sample exhibited improvement after the intervention, which was evident through the raw scores of the test for each individual. Notably, in one of the patients, their score became three times lower than the value recorded before the intervention. Although the intervention did not specifically target depressive symptoms, several hypotheses can be posited to explain this outcome. Firstly, the weekly interaction with other participants and the researchers themselves likely played a significant role, with social engagement, particularly during the pandemic, possibly contributing to the reduction of these symptoms. Additionally, engaging in activities during the sessions and completing homework assignments may have offered moments of leisure, even though the primary aim was rehabilitation. Furthermore, the participants’ self-perceived clinical improvements in cognitive domains may have also influenced the reduction of depressive symptoms. A cited study supports these findings, as it indicated that cognitive training held promise in addressing learning, memory, executive functions, activities of daily living, general cognitive issues, depression, and self-assessment^24^, aligning with the outcomes of this study.

In light of the findings from this research, there arises a question as to why the rehabilitative effect was predominantly observed in the domain of alternating attention, as opposed to other forms of attention. The literature has closely linked alternating attention with mental flexibility, wherein it is conceptualized as the ability to switch responses between stimuli, as exemplified by the Trail Making Test — Part B^25^ Neuroplasticity emerges as a pivotal factor in the gradual recovery of brain function following a stroke, potentially playing a more pronounced role in the superior colliculus—a brain region primarily responsible for shifting attentional focus toward anticipated stimuli, a fundamental component of alternating attention^26^. This disparity may offer a plausible explanation for the noteworthy improvement observed specifically in this particular facet of attention^27^.

While certain data from the present study did not yield statistically significant differences, notable clinical improvements were observed in some participants. For instance, with regard to the ability of alternating attention, two patients who initially exhibited moderate and severe impairments in Part B of the Trails Test demonstrated post-intervention improvements that indicated clinical progress toward preserved and moderate performance, respectively. Thus, although these improvements did not reach statistical significance, which may have been influenced by the study’s limited sample size, the clinical amelioration holds significant importance in the daily lives of these individuals. It enhances their independence and bolsters their sense of self-efficacy, as these improvements were personally perceived and reported by the participants themselves.

Another illustrative case pertains to the outcomes related to the skill of selective attention, gauged through the Stroop effect. It was discerned that, at the outset (T1), five patients already demonstrated preserved performance, with only one participant exhibiting moderate impairment. Subsequently, at T2, those participants with preserved performance sustained their proficiency in selective attention. Conversely, the sole individual who displayed impairment at T1 exhibited clinical improvement at T2, achieving performance levels equivalent to the healthy population’s average. In essence, this participant experienced clinical improvement, with the complete remission of the symptom.

While, up to the time of composing this study, we were unaware of other publications assessing the efficacy of remotely administered cognitive rehabilitation, it is worth noting a study conducted by Schoenberg et al. in 2008^28^. In their study, they compared online teletherapy with face-to-face teletherapy and found that digital teletherapy appears to be a feasible option for individuals with acquired brain injuries. Nevertheless, the authors emphasize that their study should not be construed as advocating teletherapy over in-person rehabilitation services, underscoring the necessity for further research on this topic.

Another study evaluated the impact of cognitive training on healthy elderly individuals and concluded that the benefits were on par with those achieved through face-to-face training, underscoring its potential as a viable public health intervention^29^. However, it is crucial to emphasize that 40% of the study’s participants had attained higher education, possessed access to both a computer and internet at home, and were generally in good health. These factors can be viewed as facilitators for online cognitive training, in contrast to our sample, where only one participant had completed higher education, and all had received a recent diagnosis of stroke.

All the sessions were conducted at the outset of the COVID-19 pandemic, a time when most participants were practicing isolation, primarily due to their belonging to a high-risk group. Consequently, they reported limited social interactions during this period and displayed notable engagement and commitment during the meetings. It is therefore plausible to consider that the weekly social contact with the researchers may have also contributed to the improvement in depressive symptoms, as reflected in the levels of depressive symptomatology. Beyond the quantitative analysis of the administered instruments, it is noteworthy that the participants themselves reported enhanced mood, heightened motivation in engaging with others, and a personal perception of cognitive improvement.

A study exploring group treatment strategies has indicated their capacity to optimize therapeutic elements, encompassing universality, altruism, the nurturing of hope, and mutual support. These elements are underpinned by participants’ ability to identify with one another concerning their unique grievances and requirements^30^. Another study, which employed game-based training, yielded outcomes akin to those observed in the current investigation^31^. The authors of the referenced study identified a statistically significant difference in the Trails Test - Part B when comparing pre- and post-intervention scores. Conversely, in the assessment of sustained attention, no statistically significant difference was observed. These findings align with the outcomes presented in the current study. Furthermore, there is a notable similarity in the methodology employed in both studies, with each comprising 15 sessions conducted over a period of 15 weeks, one session per week, with each session spanning approximately 45 minutes. Additionally, there is a close resemblance in the mean age of the participants in both studies, with an average of 67.7±7.1 years in the study by Martel et al.^31^ and 65±7.79 years in the present study.

A study assessed sustained and alternating attention using the identical instruments employed in the current study, namely the Trail Making Test — Part A and Trail Making Test — Part B, respectively^32^. The authors observed that participants required more time to complete Part B of the test, which assesses alternating attention, in comparison to Part A, which evaluates sustained attention. This disparity underscores the individuals’ necessity for a longer pause when shifting their attention repetitively compared to maintaining focus over time. Consequently, these results substantiate previous findings suggesting that Part B entails greater motor and perceptual complexity relative to Part A. Additionally, owing to the alternating command between two sequences, Part B necessitates a heightened demand on attention and working memory.

In conclusion, it is evident that clinical improvement holds significant importance in the daily lives of these individuals, enhancing their independence and reinforcing their sense of self-efficacy, as personally perceived and reported by them. This improvement also extends to the mitigation of depressive symptoms. However, it is worth noting that rehabilitation yielded a statistically significant difference in only one type of attention. To delve deeper into this treatment modality, further studies with a larger participant pool and the inclusion of a control group are warranted.

This study faced limitations stemming from a small sample size, primarily driven by a substantial dropout of participants necessitated by the transition to a remote model induced by the COVID-19 pandemic. Patients were initially recruited from public hospitals, and a significant proportion lacked internet access at home, rendering their participation infeasible. Additionally, the requirement for in-person administration of pre- and post-intervention tests posed challenges in recruiting new patients during a certain phase of the pandemic. Many individuals were observing strict social isolation measures and relied on public transportation, further complicating their engagement.

Moreover, the online format posed an additional challenge, as the majority of participants were not accustomed to using computers and mobile phones, often requiring assistance from third parties. Lastly, our analysis was constrained by the absence of information regarding the specific types of strokes, the extent of lesions, and their precise locations, limiting the depth of our evaluation.
